# Ever-increasing viral diversity associated with the red imported fire ant *Solenopsis invicta* (Formicidae: Hymenoptera)

**DOI:** 10.1186/s12985-020-01469-w

**Published:** 2021-01-06

**Authors:** César Augusto Diniz Xavier, Margaret Louise Allen, Anna Elizabeth Whitfield

**Affiliations:** 1 Department of Entomology and Plant Pathology, North Carolina State University, 840 Main Campus Drive, Raleigh, NC 27606 USA; 2 U. S. Department of Agriculture, Agricultural Research Service, Biological Control of Pests Research Unit, 59 Lee Road, Stoneville, MS 38776 USA

**Keywords:** Red imported fire ant, *S. invicta*, Virus diversity, Virome, Metatranscriptome, *Tenuivirus*, *Mononegavirales*, *Bunyavirales*

## Abstract

**Background:**

Advances in sequencing and analysis tools have facilitated discovery of many new viruses from invertebrates, including ants. *Solenopsis invicta* is an invasive ant that has quickly spread worldwide causing significant ecological and economic impacts. Its virome has begun to be characterized pertaining to potential use of viruses as natural enemies. Although the *S. invicta* virome is the best characterized among ants, most studies have been performed in its native range, with less information from invaded areas.

**Methods:**

Using a metatranscriptome approach, we further identified and molecularly characterized virus sequences associated with *S. invicta*, in two introduced areas, U.S and Taiwan. The data set used here was obtained from different stages (larvae, pupa, and adults) of *S. invicta* life cycle. Publicly available RNA sequences from GenBank’s Sequence Read Archive were downloaded and de novo assembled using CLC Genomics Workbench 20.0.1. Contigs were compared against the non-redundant protein sequences and those showing similarity to viral sequences were further analyzed.

**Results:**

We characterized five putative new viruses associated with *S. invicta* transcriptomes. Sequence comparisons revealed extensive divergence across ORFs and genomic regions with most of them sharing less than 40% amino acid identity with those closest homologous sequences previously characterized. The first negative-sense single-stranded RNA virus genomic sequences included in the orders *Bunyavirales* and *Mononegavirales* are reported. In addition, two positive single-strand virus genome sequences and one single strand DNA virus genome sequence were also identified. While the presence of a putative tenuivirus associated with *S. invicta* was previously suggested to be a contamination, here we characterized and present strong evidence that Solenopsis invicta virus 14 (SINV-14) is a tenui-like virus that has a long-term association with the ant. Furthermore, based on virus sequence abundance compared to housekeeping genes, phylogenetic relationships, and completeness of viral coding sequences, our results suggest that four of five virus sequences reported, those being SINV-14, SINV-15, SINV-16 and SINV-17, may be associated to viruses actively replicating in the ant *S. invicta*.

**Conclusions:**

The present study expands our knowledge about viral diversity associated with *S. invicta* in introduced areas with potential to be used as biological control agents, which will require further biological characterization.

## Background

Insects are the most abundant and diverse group of animals on earth [1]. High throughput sequencing has led to huge advances in revealing previously unknown diversity of insect viruses, significantly contributing to filling deep phylogenetic gaps along evolutionary history within the most diverse viral lineages [2–4]. Nonetheless, like insect diversity, the diversity of viruses associated with insects is far from clear [1, 3]. While many studies have focused on viromes of arbovirus-transmitting insects, especially those involved in transmission of medically important viruses, such as mosquitoes [5–8], other groups that are important pests either impacting agricultural or natural ecosystems, such as invasive ants, have been less studied [9–11]. In addition to contributing to better understanding of basic aspects of virus ecology and evolution, these studies may contribute to new opportunities to use viruses as tools to develop more sustainable insect control methods [12–14].

The red imported fire ant, *Solenopsis invicta*, is an invasive pest causing significant ecological impact and economic loss in invaded areas [15, 16]. Originating from South America, *S. invicta* was accidentally introduced into the southern region of the United States (U.S.) almost a century ago, becoming a serious problem [17]. Since then, it has spread throughout the southeastern U.S. and more recently into Oklahoma, New Mexico, Arizona, and California [18]. Limited introduction events, likely associated with small founder populations, led to a significant reduction in natural enemies and enemy diversity associated with *S. invicta* in introduced areas [19–21]. Therefore, *S. invicta* populations may reach sizes even greater than those observed in its native range, making control difficult and even more costly [22]. High densities observed in *S. invicta* populations in the U.S. have facilitated its dispersal across the world, contributing to repeated introduction events in several countries, such as China, Taiwan, and Australia [18]. Morrison et al. [23] demonstrated, based on predictive models, that most tropical and subtropical regions worldwide are potentially appropriate for *S. invicta* infestation. Highly competitive ability, generalist feeding habits and high populations densities make *S. invicta* a successful invasive species causing huge disturbance in biodiversity by displacing native ants and other arthropods in introduced regions [15]. Currently, chemical insecticides are the most common control strategy used against *S. invicta* [24]. Low efficacy due to temporary effects of chemical applications, high cost in extensive areas, and off-target effects harmful to beneficial and other native species are substantial impairments to addressing invasive ant damage and expansion. Therefore, establishment of management strategies that are both environmentally friendly and self-sustainable are necessary.

Classical biological control has been one strategy used in an attempt to control this pest in the U.S., with viruses considered a promising resource to be used as biopesticides [24–26]. Over the last decade, a great effort has been made in characterizing the *S. invicta* virome pertaining to potential use in biological control [11, 27–30]. To date, the *S. invicta* virome is composed of mainly positive-sense single-strand RNA (+ ssRNA) viruses in the order *Picornavirales*. These include eleven viruses from families *Dicistroviridae*, *Polycipiviridae*, *Iflaviridae*, *Soliniviridae* and two unclassified viruses [11, 31]. Additionally, one double-strand RNA (dsRNA) and one single-strand DNA (ssDNA) of the families *Totiviridae* and *Parvoviridae*, respectively, have been characterized [11, 32]. While most of these viruses have been reported associated with *S. invicta* in its native range, only the species *Solenopsis invicta virus 1*, *Solenopsis invicta virus 2*, *Solenopsis invicta virus 3*, *Solenopsis invicta virus 6* and *Nylanderia fulva virus 1* have been reported in the United States [11, 31]. Although great progress has been reached in identification and molecular characterization of the *S. invicta* virome, the effect of these viruses and their potential use in biological control will require additional investigation. Interactions among *S. invicta* and Solenopsis invicta virus 1 (SINV-1), SINV-2 and SINV-3, have been the only ones previously characterized [25, 33, 34]. SINV-1 was shown to affect claustral queen weight making them lighter than uninfected ones, whereas SINV-2 directly affected fitness of queens by reducing their reproductive output [33]. SINV-3 was shown to be the most aggressive virus, causing significant mortality in *S. invicta* colonies, with greatest potential as a biological control agent [25, 35].

Here using a metatranscriptome approach, we identified and molecularly characterized five new virus genome sequences associated with *S. invicta* in two introduced areas, U.S and Taiwan. These investigations utilized existing publicly available RNA sequences deposited in NCBI GenBank as Sequence Read Archive (SRA) data files (Table 1). Five new virus sequences were found associated with *S. invicta*, with the first negative-sense single-stranded RNA (-ssRNA) virus sequences included in the orders *Bunyavirales* and *Mononegavirales* reported. In addition, two + ssRNA viruses sequences, included in the family *Iflaviridae* and an unassigned species, and a partial ssDNA virus sequence, were also characterized.

**Table 1:** Information summary of *S. invicta* transcriptome dataset analyzed in this study

Sample code	SRA access #	Stage	Part of insect body	Origin	Date of collection	Other information	References
A	SRX3035962	Larvae	Whole body	Mississippi, USA	March 2016	Polygynous	Allen et al. [36]
B	SRX3035961	Larvae	Whole body	Mississippi, USA	March 2016	Polygynous	Allen et al. [36]
C	SRX3035960	Larvae	Whole body	Mississippi, USA	March 2016	Polygynous	Allen et al. [36]
X	SRX3035959	Pupa	Whole body	Mississippi, USA	March 2016	Polygynous	Allen et al. [36]
Y	SRX3035964	Pupa	Whole body	Mississippi, USA	March 2016	Polygynous	Allen et al. [36]
Z	SRX3035963	Pupa	Whole body	Mississippi, USA	March 2016	Polygynous	Allen et al. [36]
Q2	DRX037806	Adults	Whole body	Texas, USA	September 2012	Polygynous, queen	Morandin et al. [37]
W2	DRX037809	Adults	Whole body	Texas, USA	September 2012	Polygynous, worker	Morandin et al. [37]
W3	DRX037810	Adults	Whole body	Texas, USA	September 2012	Polygynous, worker	Morandin et al. [37]
Y05	SRX5464977	Adults	Brain	Florida, USA	May 2014	Queen	–*
K05	SRX5464984	Adults	Brain	Florida, USA	May 2014	Queen	–
CA01	SRX5464990	Adults	Brain	Florida, USA	May 2014	Queen	–
2-small	SRX5822389	Adults	Whole body	Taiwan	May 2014	A pool of two virgin queens, lab colony	Fontana et al. [38]

*Reference is not available yet

## Methods

### Data set selection and sequencing

Contigs corresponding to putative new virus genome sequences were initially obtained from a transcriptome project studying differential gene expression between larvae and pupae stages of *S. invicta* collected in Mississippi, U.S. [36]. To further investigate and characterize viral diversity associated with *S. invicta*, six libraries [36] were downloaded from GenBank’s Sequence Read Archive (SRA) and analyzed. In addition, to determine whether those viruses were present in other locations in U.S. and abroad, several transcriptome libraries deposited at SRA were compared against those previous putative viral contigs using BLASTn. Libraries that presented high abundance of reads mapping to putative viral contigs with an E-value cutoff lower than 1e^−2^ were downloaded and further analyzed. The final data set analyzed here consisted of 13 libraries, all from *S. invicta* transcriptomes. Detailed information on the samples are presented in Table 1. All information about sample collection, RNA extraction, library preparation and high throughput sequencing has been previously described [36–38].

### De novo assembly and virus genome characterizations

Sequencing reads were trimmed for quality (Additional file 1: Table S1) and de novo assembled using CLC Genomics Workbench 20.0.1 with default settings. Reads associated to *S. invicta* were first filtered by mapping them back to the *S. invicta* genome (GenBank accession: GCA_000188075.2) and only unmapped reads were used for de novo assembling (Additional file 1: Table S1). Contigs were compared against NCBI non-redundant protein sequence (nr) using BLASTx and those predicted to contain near full-length and intact coding sequences homologous to viral sequences previously described were confirmed by mapping reads back to obtain consensus sequences for each library. Potential open reading frames (ORFs) of the putative new viruses were predicted using ORF finder (NCBI) and by comparative analysis with those related viruses. For identification of conserved functional domains, a domain-based BLAST search was performed against the Conserved Domain Database (CDD). ORFs that did not present any conserved domain were predicted based on comparative analysis with other known closely related viruses [5].

### Multiple sequence alignment and phylogenetic analysis

For phylogenetic analyses, one representative sequence for each taxon most closely related to viruses described here was retrieved from GenBank according to BLASTx analysis. ORF integrity was checked and extracted using ORF finder and the presence of conserved domain characteristics for each group was checked at CDD before proceeding to phylogenetic analysis. For highly divergent data sets, regions comprising protein conserved domains were extracted using a script written in R software according to coordinates obtained from CDD for each sequence, and only the conserved domain was used (as indicated below in the figure legends). Multiple sequence alignments of deduced amino acid sequences were prepared using the MUSCLE option in MEGA7 [39]. Alignments were manually checked and adjusted when necessary. Phylogenetic trees were constructed using Bayesian inference performed with MrBayes 3.2.6 [40]. Estimates of the amino acid model were automatically conducted by setting the prior for the amino acid model to mixed, as implemented in MrBayes. The analyses were carried out running two independent runs of 20,000,000 generations with sampling at every 1000 generations and a burn-in of 25%. Convergence between runs were accepted when average standard deviations of split frequencies was lower than 0.01. Trees were visualized and edited using FigTree and Corel Draw, respectively.

### Inferring viruses-*S. invicta* association

Most samples used here were prepared from whole bodies of ants, and to differentiate truly replicating viruses from those that might be from food contamination or commensal organisms, viral abundance was compared with abundance of three housekeeping genes: cytochrome c oxidase subunit I (*cox1*), that presented high transcriptional levels, ribosomal protein L18 (*rpl18*) and translation elongation factor 1 (*eif1-beta*), both with lower transcriptional levels. Viral and internal genes abundance were calculated as percentage of viral or housekeeping reads per total number of reads in each library (Table 2). Differences among groups was assessed using non-parametric Kruskal–Wallis test followed by post hoc multiple comparison test using Fisher’s least significant difference [41] using Agricolae package [42] in R software. The data were log-transformed before statistical testing. Thus, a virus sequence was suggested to be from a virus hosted by *S. invicta* if they qualified in at least two of the criteria as previously suggested by [8] with slight modifications: (i) virus abundance was within the range or higher abundance than housekeeping genes; (ii) viral reads per total number of reads in a library was higher than 0.01%; (iii) they were phylogenetically close to another insect virus; and (iv) complete viral coding sequence regions were recovered.

**Table 2:** Abundance of viruses and housekeeping genes, cytochrome c oxidase subunit I (*cox1*), ribosomal protein L18 (*rpl18*) and translation elongation factor 1 (*eif1-beta*), expressed in percentage of viral reads per total number of reads in each library

Sample	SRA access #	Virus abundance (%)^*^									Housekeeping genes (%)		
		SINV-14					SINV-15	SINV-16	SINV-17	SINaDNV	*cox1*	*rpl18*	*eif1-beta*
		RNA1	RNA2	RNA3	RNA4	Total^&^							
A	SRX3035962	–	–	–	–	–	0.008	**0.516**	**0.128**	0.002	0.3910	0.0238	0.0163
B	SRX3035961	**0.047**	0.019	**0.042**	**0.072**	**0.180**	0.013	**0.327**	**0.235**	–	0.7102	0.0263	0.0267
C	SRX3035960	**0.046**	0.005	**0.051**	**0.048**	**0.150**	0.019	**0.120**	**0.281**	0.002	0.4324	0.0246	0.0305
X	SRX3035959	–	–	–	–	–	**0.009**	–	–	–	0.2860	0.0161	0.0080
Y	SRX3035964	–	–	–	–	–	**0.023**	–	–	–	0.3236	0.0153	0.0089
Z	SRX3035963	**0.374**	**0.032**	**0.131**	**0.089**	**0.626**	**0.039**	–	–	–	0.2687	0.0135	0.0090
Q2	DRX037806	**1.469**	**0.055**	**0.240**	**0.944**	**2.708**	**0.211**	–	–	–	1.0036	0.0530	0.0469
W2	DRX037809	**1.679**	0.006	**0.088**	**0.601**	**2.374**	**0.020**	–	–	–	2.1720	0.0342	0.0113
W3	DRX037810	**1.658**	0.006	**0.089**	**0.619**	**2.372**	**0.020**	–	–	–	1.6576	0.0351	0.0117
Y05	SRX5464977	**6.803**	**0.941**	**0.650**	0.001	**8.394**	–	–	–	–	0.4903	0.0119	0.0048
K05	SRX5464984	–	–	–	–	–	–	–	–	–	0.4957	0.0141	0.0046
CA01	SRX5464990	–	–	–	–	–	–	–	–	–	0.4639	0.0158	0.0064
2-Small	SRX5822389	**0.084**	0.009	0.017	0.019	**0.130**	–	–	–	–	0.9619	0.0712	0.0340

^*^Viruses with abundance equal or higher than those shown by at least one of the housekeeping genes are highlighted in bold and those with complete or nearly-complete genomes are underlined

^&^Total: means all segments together

### Principal component analysis (PCA) of compositional bias

Synonymous codon usage (SCU) and dinucleotide composition bias has been used to infer putative host-virus association and taxonomic placement for a given virus sequence [43, 44]. To gain further insight about the host of the tenui-like sequences reported here, we used principal component analysis (PCA) to compare the compositional bias between plant tenuivirus and other viruses infecting plants and animals (vertebrates and invertebrates). SCU counts were determined for the full-length nucleotide sequence of RNA-dependent RNA polymerase (RdRp), glycoprotein, nucleocapsid and non-structural protein 4 (NS4) protein, using the coRdon package [45] in R software. Dinucleotide bias was calculated as the frequency of dinucleotide *XY* divided by the product of frequencies of nucleotide *X* and nucleotide *Y* using the SeqinR package [46] in R software. Sequences highly divergent with no conserved domain according to CDD analysis were not included. Principal components analysis was performed on codon usage counts and dinucleotide bias using the function prcomp and plotted using the factoextra package [47], both implemented in R software.

## Results

### New virus sequences found associated with *S. invicta* transcriptome

**Fig. 1:**
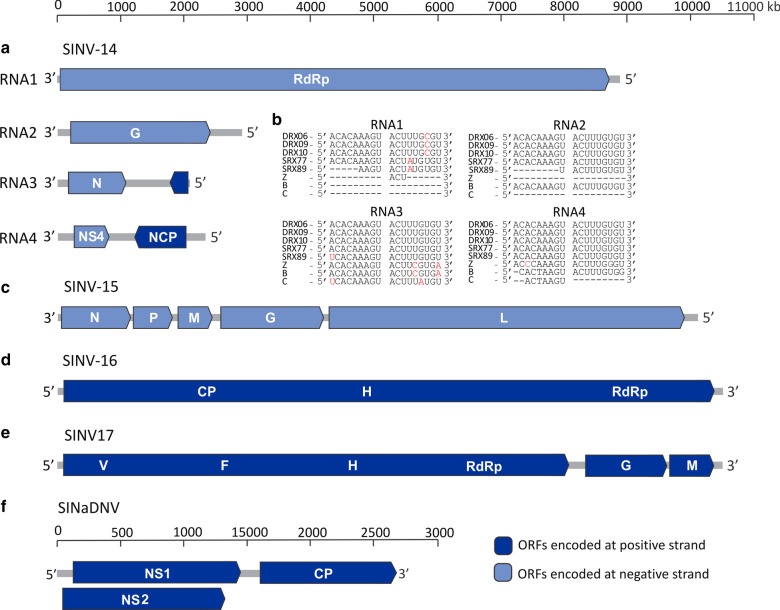
Genome organization of five putative new viruses associated with *S. invicta* transcriptomes. Predicted open reading frames (ORFs) encoding proteins homologous to known viral proteins or conserved domain that they encode are represented and colored according to their orientation in the genome (dark blue: those encoded on positive strand and light blue: those encoded on negative strand). Graphic representation of **A** segmented negative single-strand RNA genome S. invicta virus 14 (SINV-14) and **B** conserved consensus sequence located at the ends of tenuivirus genome segment (5′-ACACAAAGU and ACUUUGAGU-3′) detected in SINV-14 genome. Dashed lines indicate incomplete sequence. Nucleotides highlighted in red differ from consensus sequence. **C** Negative-sense single-strand RNA genome S. invicta virus 15 (SINV-15), and positive-sense single-strand RNA genomes **D** S. invicta virus 16 (SINV-16) and **E** S. invicta virus 17 (SINV-17) are shown. **F** Partial double-strand DNA genome of *S. invicta*-associated densovirus (SINaDNV) is represented. RdRp and L: RNA-dependent RNA polymerase; G: glycoprotein; N: nucleocapsid protein; p3: unknown; NS4: non-structural protein 4; NCP: non-nucleocapsid protein; P: phosphoprotein; M: matrix protein; CP: capsid protein; H: Helicase; V: viral methyltransferase; F: Ftsj-like methyltransferase; NS1and NS2: non-structural protein 1 and 2, respectively

Virus sequences were found associated with *S. invicta* transcriptomes collected from three geographic locations in the U.S. and one location from Taiwan, representing different stages of the *S. invicta* life cycle (Table 1). De novo assembling of non-host reads (Additional file 1: Table S1) from 13 libraries followed by BLAST analyses revealed the presence of complete or near-complete genomes sequences of 4 putative new single-strand RNA (ssRNA) viruses, tentatively named S. invicta virus 14 (SINV-14), SINV-15, SINV-16 and SINV-17, and one partial genome sequence encompassing the almost full-length coding sequence of a putative ssDNA virus, named S. invicta-associated densovirus (SINaDNV; Fig. 1). Sequence comparisons revealed extensive divergence across ORFs and genomic regions with most of them sharing less than 40% amino acid identity compared to those closest homologous sequences previously characterized (Additional file 2: Table S2). In addition, typical domains associated with RdRp and other proteins involved in virus replication, encapsidation, and entry into the cell were found and characteristic of known virus groups, again supporting the viral identity of those sequences (Additional file 3: Table S3). Further analyses demonstrated that SINV-14 and SINV-15 fit within the order *Bunyavirales* and *Mononegavirales*, respectively (Fig. 1). The two other + ssRNA genome sequences, SINV-16 and SINV-17, were included within the family *Iflaviridae* and into an unclassified group closely related to a proposed new genus of insect-infecting viruses “*Negevirus*” [48], respectively. Finally, the partial ssDNA virus sequence SINaDNV was included into family *Parvoviridae* subfamily *Densovirinae*.

### Molecular and phylogenetic characterization of new viruses

#### Negative-sense RNA viruses

The SINV-14 genome sequence consists of a linear, negative-sense single-strand RNA composed of four segments, with similar organization of typical plant tenuiviruses (Fig. 1A). A domain-based BLAST search was performed confirming the presence of conserved domains from typical members of family *Phenuiviridae* for RdRp, glycoprotein, putative NS4 and non-nucleocapsid protein (Additional file 3: Table S3). Identity analysis of deduced amino acid sequences of coding regions across all segments demonstrated an extensive divergence with those most closely related viruses (Additional file 2: Table S2). While most ORFs were most similar to that of tenui-like virus Fitzroy Crossing tenui-like virus 1 (FCTenV1) and otter fecal bunyavirus, the ORF encoded in the positive strand of RNA4 was related to those of non-nucleocapsid proteins from plant tenuiviruses (Additional file 2: Table S2). Interestingly, the ORF encoded on the negative strand of RNA4 contained the specific domain of movement proteins (pfam03300) of plant tenuiviruses (Additional file 3: Table S3). Further sequence analysis revealed the presence of conserved sequences at the ends of all four segments identical to those found at tenuivirus genomes (3′-ACUUUGUGU and ACACAAAGU-5′; Fig. 1B) [49], indicating that full length genome segments were likely assembled from the RNA-Seq datasets.

**Fig. 2:**
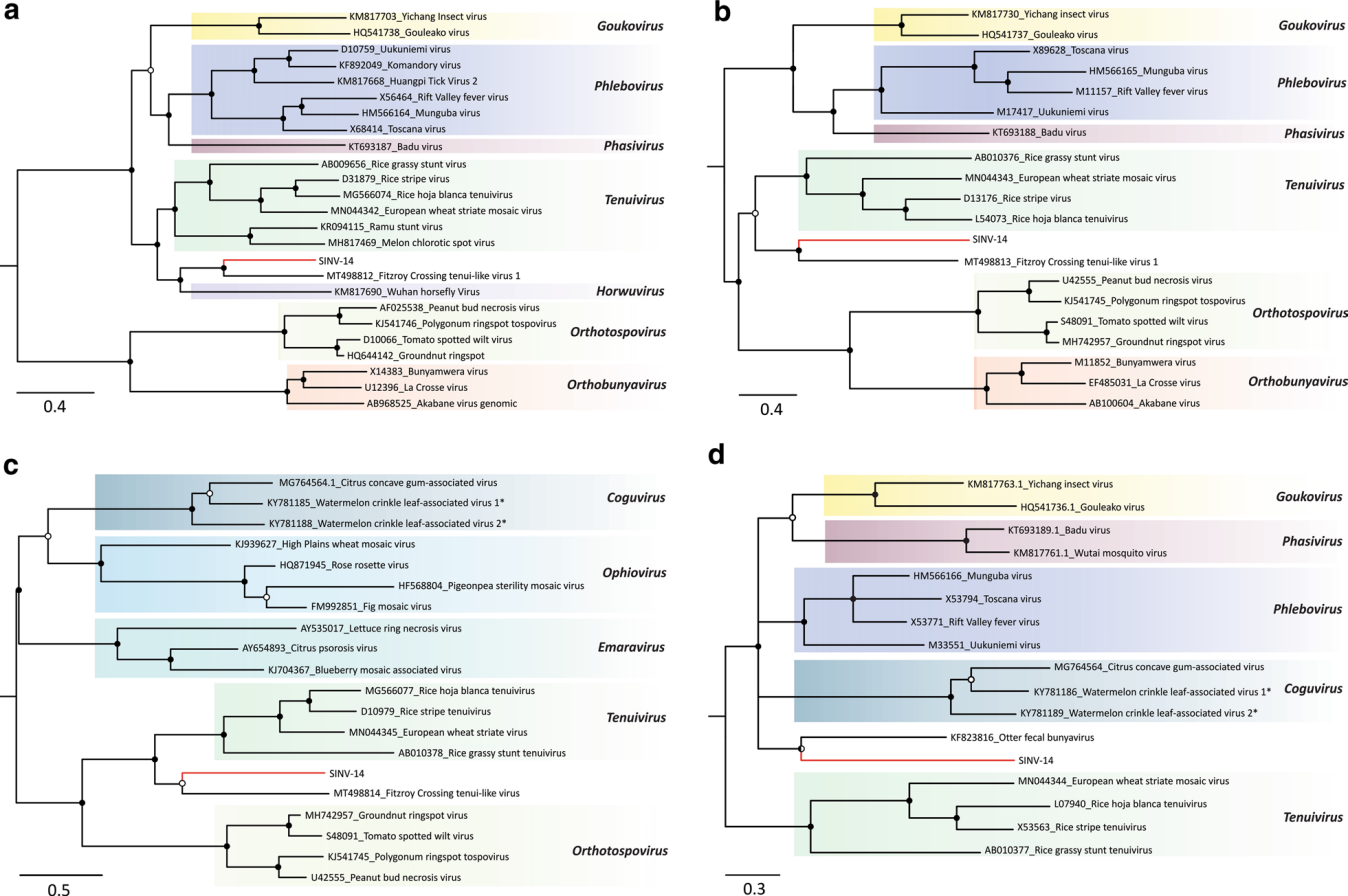
Evolutionary history inferred for different genomic segments of S. invicta virus 14 (SINV-14). Midpoint-rooted Bayesian phylogenetic trees based on **A** full-length amino-acid sequences of RNA-dependent RNA polymerase (RdRp), **B** amino-acid sequence of conserved glycoprotein domain, **C** full-length amino-acid sequence of movement protein and **D** full-length amino-acid sequence of nucleocapsid protein. Bayesian posterior probabilities are shown at the nodes. Nodes with posterior probability values between > 0.50 and ≤ 85 are indicated by empty circles, > 0.85 and < 0.95 are indicated by half-filled circles and those with values ≥ 0.95 are indicated by filled circles. The scale bar at the bottom indicates the number of amino acid substitutions per site. Branches highlighted in red indicate virus characterized in this study. Genera are indicated at the right side. Proposed species unaccepted yet by International Committee on Taxonomy of Viruses (ICTV) are followed by an asterisk. Proteins sequences encoded by cognates components for a given virus that do not have conserved domain according to Conserved Domain Database (CDD) were not included

To further determine the taxonomic placement of SINV-14 within the order *Bunyavirales*, Bayesian phylogenetic trees were inferred for representative ORFs from all four segments (Fig. 2). In accordance with identity analysis, SINV-14 RdRp was most closely related to that of FCTenV1 (Fig. 2A). These two viruses clustered closely to the tenui-like *Horsefly horwuvirus* (Wuhan horsefly virus, WhHV), the only species within the genus *Horwuvirus* (Fig. 2A). Moreover, SINV-14 glycoprotein and NS4 phylogenetic trees were congruent with RdRp, clustering closest to FCTenV1 and composing a sister clade with typical plant tenuiviruses (Fig. 2B and C). Interestingly, the phylogenetic tree of the nucleocapsid protein was incongruent compared to those of other viral proteins (Fig. 2D). It was more closely related to that of otter fecal bunyavirus and other representative viruses within family *Phenuiviridae* rather than plant tenuiviruses (Fig. 2D), in accordance with the identity analysis (Additional file 2: Table S2).

**Fig. 3:**
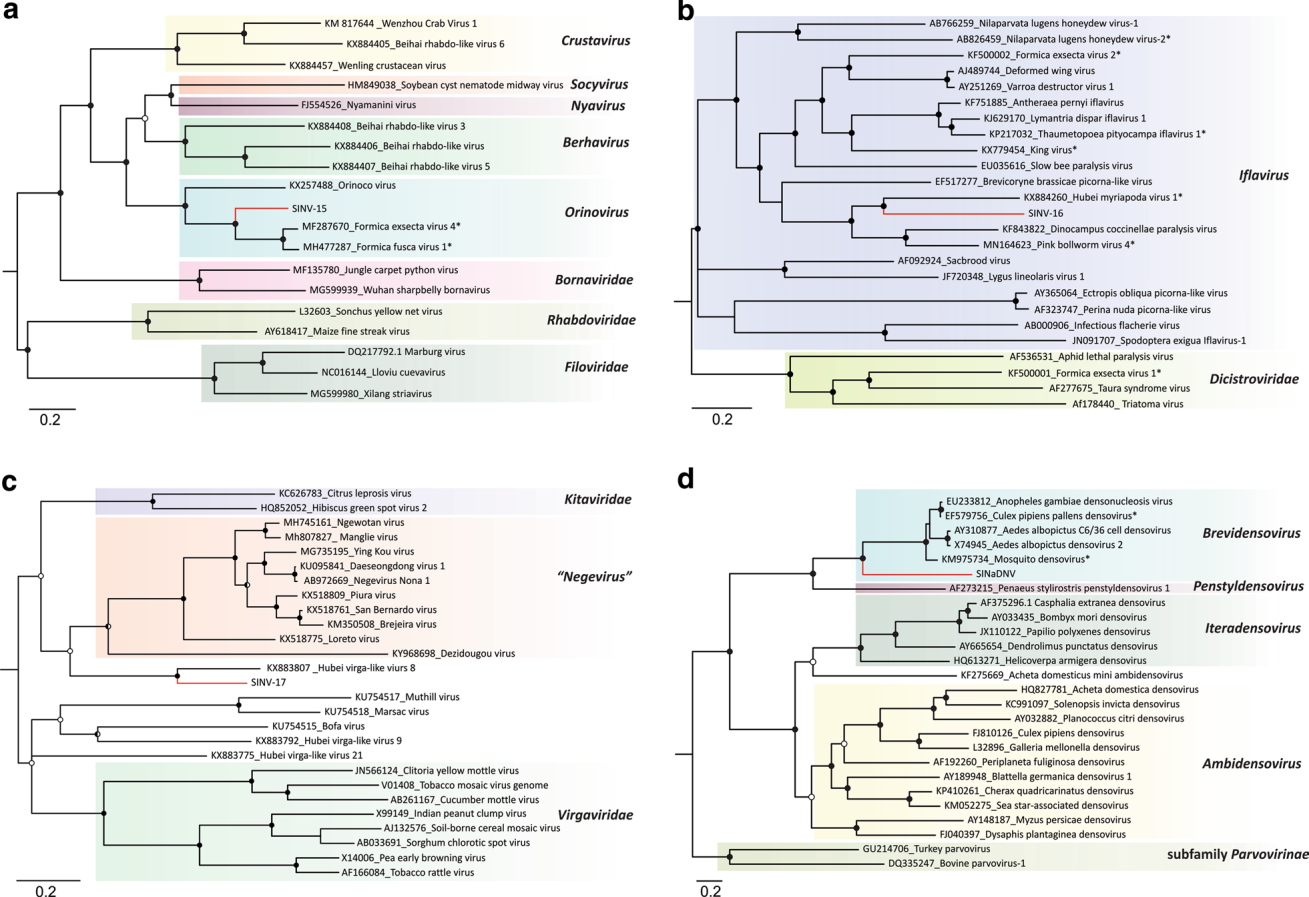
Evolutionary history of new viruses associated to *S. invicta* transcriptome based on Bayesian inference. Midpoint-rooted phylogenetic trees were constructed based on **A** conserved domain (pfam00946) of RNA-dependent RNA polymerase (RdRp) of S. invicta virus 15 (SINV-15) and representative species of the order *Mononegavirales*, **B** conserved domain (pfam00680) of RdRp of S. invicta virus 16 (SINV-16) and representative species of the families *Iflaviridae* and *Dicistroviridae* and **C** full-length amino-acid sequences of RdRp of S. invicta virus 17 (SINV-17) and representative species of the proposed new genus *Negevirus* (unassigned to any family) and families *Virgaviridae* and *Kitaviridae*, are shown. **D** Bayesian phylogenetic tree based on full-length amino-acid sequences of non-structural gene 1 (NS1) of S. invicta-associated densovirus (SINaDNV) and representative species of the family *Parvoviridae*. Bayesian posterior probabilities are shown at the nodes. Nodes with posterior probability values between > 0.65 and ≤ 85 are indicated by empty circle, > 0.85 and < 0.95 are indicated by half-filled circles and those with values ≥ 0.95 are indicated by filled circles. The scale bar at the bottom indicates the number of amino acid substitutions per site. Branches highlighted in red indicates viruses characterized in this study. Genera/Families/Subfamilies are shown at right side

The SINV-15 genome comprises a linear negative-sense, single strand RNA varying from 10,113 to 10,135 nt (Fig. 1C), showing a typical organization of viruses within order *Mononegavirales* (Fig. 1C). Domain-based BLAST search confirmed that ORFs 1 and 5 carry nucleocapsid and RdRp conserved domains, respectively, whereas no conserved domains were identified for ORFs 2, 3 and 4 (Additional file 3: Table S3). Thus, these ORFs were inferred based on comparative analysis with other known related viruses. According to identity analysis, phylogenetic analysis based on the amino acid sequence of RdRp clustered SINV-15 closest to Formica fusca virus 1 (FfusV-1) and Formica exsecta virus 4 (FeV-4), both currently unaccepted species (Fig. 3A). The clustering of these three viruses closely related to *Orinoco virus* (ONCV), suggests that they are putative new members of this genus within the family *Nyamiviridae*.

#### Positive-sense RNA viruses

SINV-16 has a positive sense single-stranded RNA genome varying from 10,355 to 10,369 nt (Fig. 1D) with genome organization like viruses included in the order *Picornavirales*. The single ORF predicted encodes a large polyprotein (2910 amino acids) including RdRp, helicase, and the canonical picornavirus capsid protein domain (Fig. 1D and Additional file 3: Table S3). Sequence comparison revealed significant identity with two unaccepted iflavirus-related species, pink bollworm virus 4, and Hubei myriapoda virus 1 (Additional file 2: Table S2). Phylogenetic analysis of the RdRp domain clearly clustered SINV-16 within genus *Iflavirus* (Fig. 3B), most closely related to Hubei myriapoda virus 1, in a well-supported clade composed also by pink bollworm virus 4 and *Dinocampus coccinellae paralysis virus* (DcPV). These results indicate that SINV-16 is a new iflavirus reported from *S. invicta*.

The SINV-17 genome consists of a linear, positive-sense single-stranded RNA with approximately 10,341 nt predicted to encode three putative ORFs (Fig. 1E). ORF1 was predicted to encode the longest protein containing the viral methyltransferase, helicase and RdRp domains (Fig. 1E and Additional file 3: Table S3). ORF2 and ORF3 were predicted to encode structural proteins, which contain domains associated with virion glycoproteins of insect viruses and virion membrane proteins of plant and insect viruses, respectively (Additional file 3: Table S3). Interestingly, while ORFs 1 and 2 were most similar to Hubei virga-like virus 8, an unclassified virus isolated from a pool of individuals of the family Scutigeridae [3], ORF 3 showed significant identity to Loreto virus isolated from *Anopheles albimanus*, a member of the proposed genus *Negevirus* [48] (Additional file 2: Table S2). In accordance with identity analysis, SINV-17 was most closely related to Hubei virga-like virus 8, appearing to represent a sister clade to the proposed genus *Negevirus*, and possibly representing a new genus (Fig. 3C).

#### Other virus sequences

Two small contigs were found showing significant similarity with RNA virus sequences. The first one was 430 nt in length and exhibited highest identity with a hypothetical protein encoded by Beihai tombus-like virus 6 (41.13% sequence identity; 97% coverage; E-value = 4e-17; GenBank access: APG76145.1), isolated from a pool of marine crustaceans within the genus *Penaeidae* [3]. The second contig has 330 nt and showed highest identity with NfV-1 polyprotein (59.49% sequence identity; 93% coverage; E-value = 2e−4; GenBank access: AOC55078.1) from tawny crazy ant *Nylanderia fulva* [50].

In addition to the RNA viruses, we also characterized a partial genome encompassing the almost full-length coding sequence of a single-strand DNA virus (SINaDNV), predicted to encode three ORFs on the positive strand, with typical organization of viruses within the family *Parvoviridae* (Fig. 1F). The ORFs 1 and 2 were overlapped and were most similar to NS1 and NS2 of mosquito-infecting densoviruses, respectively (Additional file 2: Table S2), whereas ORF3 showed significant similarity to capsid protein of Aedes albopictus C6/36 cell densovirus (Additional file 2: Table S2). Phylogenetic analysis based on amino acid sequence of ORF1 clustered SINaDNV with viruses within subfamily *Densovirinae* and genus *Brevidensovirus* (Fig. 3D). While these results strongly suggest SINaDNV is a new densovirus species, further investigation will be needed to reveal its whole genome organization.

#### Inferring host association

**Fig. 4:**
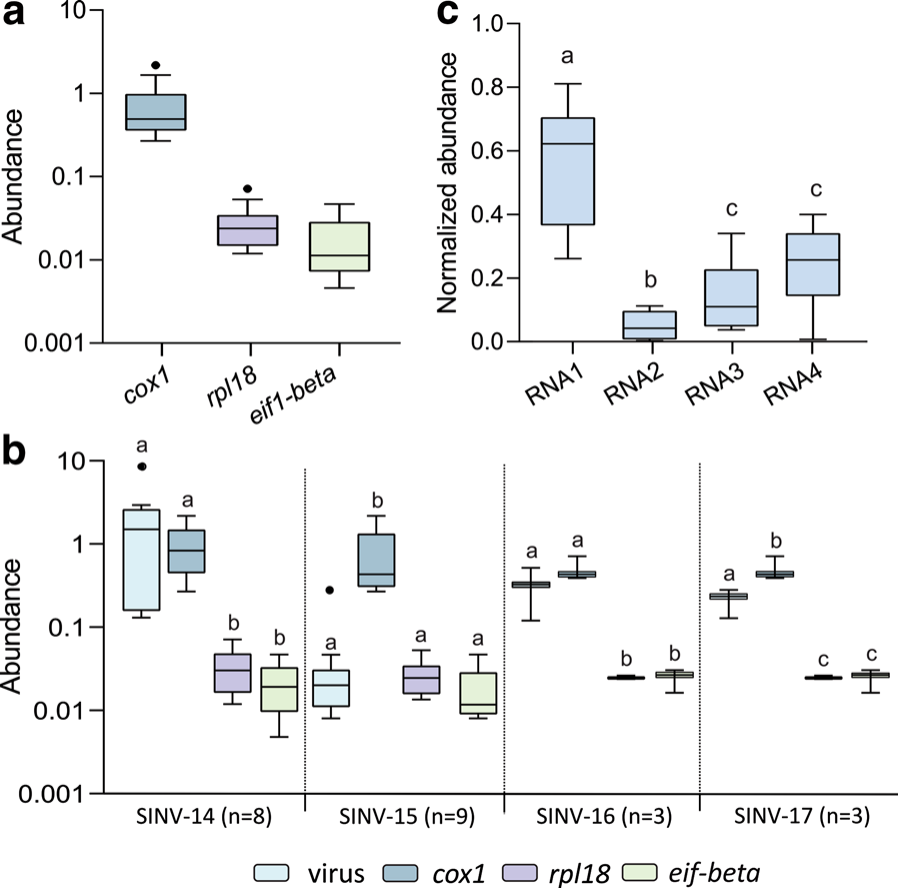
Boxplot of housekeeping genes and viruses abundance. **A** Abundance among housekeeping genes from all libraries were compared. Abundance was calculated as percentage of housekeeping or viruses reads per total number of reads in each library. **B** Abundance between viruses and housekeeping genes were compared. Statistical test was performed among virus and housekeeping genes abundance from the same libraries. **C** Normalized abundance of S. invicta virus 14 (SINV-14) segments significantly differ among them. Normalized abundance was calculated by dividing the abundance of each segment by the total abundance of the entire genome. Box plots with different letters indicate significant differences between groups according to non-parametric Kruskal–Wallis test followed by post hoc multiple comparison test using Fisher’s least significant difference (*p* < 0.05). Box plots show the first and third quartiles as a box, horizontal line corresponds to the median and whiskers correspond to 1.5 times the inter-quartile distance (IQR) from the difference between first and third quartiles

To infer host-virus association and differentiate putative viruses truly replicating from those that may be a contamination, we calculated the percentage of viral reads for each library and compared with three housekeeping genes, *cox1*, *rpl18* and *eif1-beta* (Fig. 4 and Table 2). While the abundance of all three housekeeping genes were relatively stable across different libraries, it differed among them, with *cox1* most abundant, followed by *rpl18* and *eif1-beta* (Fig. 4A). Likewise, virus abundance was relatively stable within species, except for SINV-14 that varied between 0.130 and 8.394% of viral reads compared to the total reads, considering the complete genome sequence (Table 2). For SINV-14 and SINV-16, read abundance was significantly higher than *eif1-beta* and *rpl18*, with no difference compared to *cox1* (Fig. 4B). SINV-17 abundance was significantly lower than *cox1* and higher than *eif1-beta* and *rpl18*. In contrast, SINV-15 abundance was the lowest one among them, being significantly lower than *cox1*, with no difference compared to *rpl18* and *eif1-beta* (Fig. 4B). In addition, SINV-14, SINV-15, SINV-16 and SINV-17 abundances were higher than 0.01% of reads compared to entire library (Table 2). The only exception was SINaDNV, that had a very low abundance compared to housekeeping genes and other viruses (Table 2).

Interestingly, SINV-14 abundance was much higher in a sample prepared from a dissected brain compared to those from the whole body (Tables 1 and 2). Considering the abundance of all segments, SINV-14 from the brain sample was 17-fold more abundant compared with *cox1* gene, whereas abundance of those samples prepared from whole body varied 0.135 to 2.69-fold higher than *cox1* (Table 2). Moreover, the abundance of segments was asymmetric (Fig. 4C). The reads mapping on RNA1 were significantly more abundant, followed by RNA3 and RNA4, with RNA2 the least abundant (Fig. 4C).

**Fig. 5:**
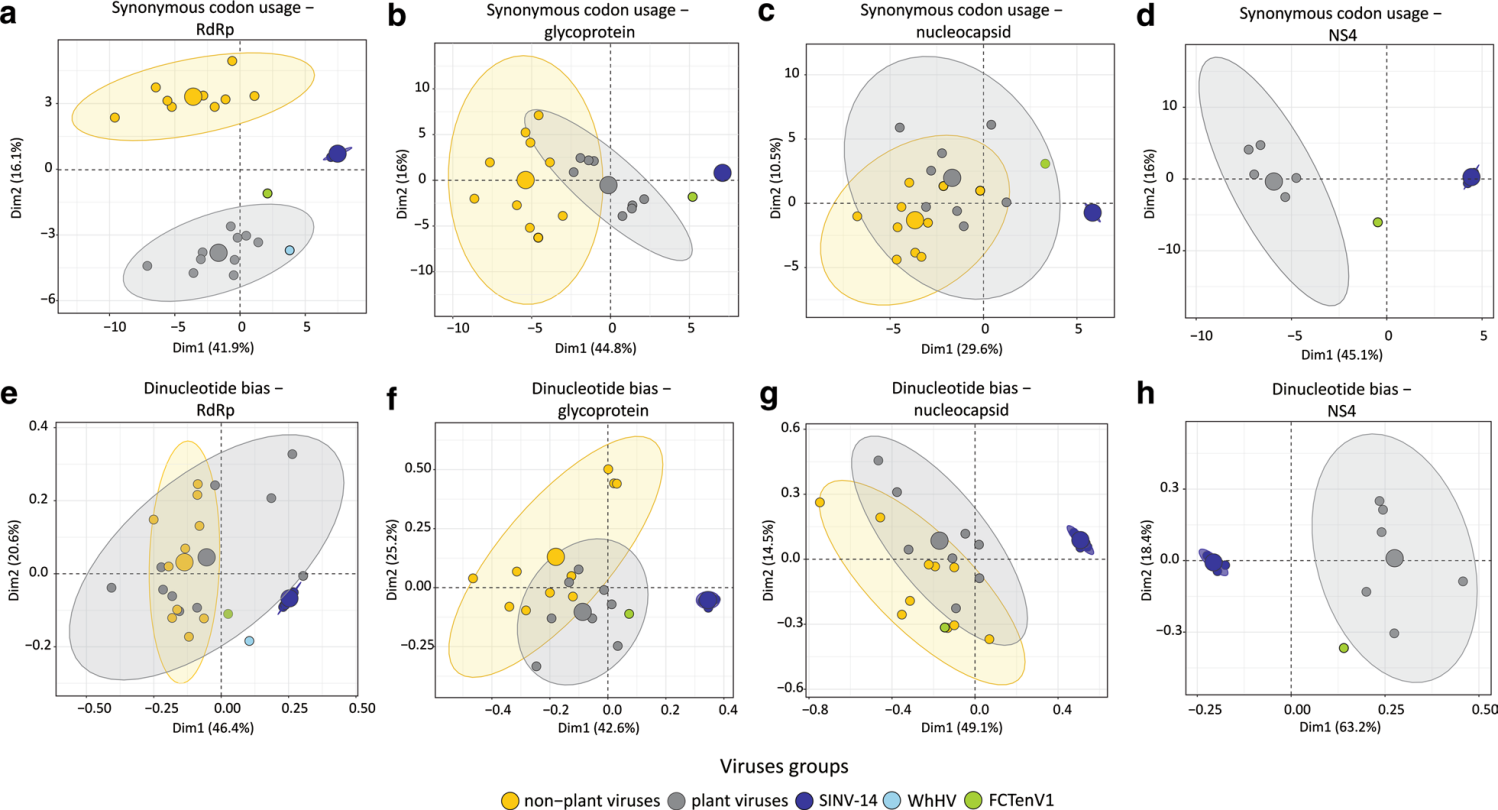
Compositional biases of Solenopsis invicta virus 14 (SINV-14) differ from plant tenuivirus and tenui-like viruses associated with flies. Principal Components Analysis (PCA) of synonymous codon usage (**A**–**D**) and dinucleotide bias (**E**–**H**) of SINV-14 and other viruses that infect plants and animals (vertebrate and invertebrate) are shown. Scatterplots show the first two principal components that account for the largest variance (shown in parenthesis) in the data set with ellipses representing each group and dots individuals. Viruses were divided into two main groups: plant viruses include those that replicate in plants and insect vectors belonging to the genera *Tenuivirus* and *Orthotospovirus* and non-plant viruses including those that do not replicate in plants belonging to the genera *Orthobunyavirus* and representatives within family *Phenuiviridae*. For detailed information of the data set see Additional file 4: Table S4. For NS4 protein, comparison was performed only for SINV-14, Fitzroy Crossing tenui-like virus1 (FCTenV1) and tenuiviruses, as this gene is not shared by other groups. WhHV, Wuhan horsefly virus

To further verify the relationship between SINV-14 and *S. invicta*, we also analyzed the codon usage and dinucleotide bias (Fig. 5). Principal component analysis of codon usage based on RdRp, glycoprotein, nucleocapsid and NS4 protein, clearly separate SINV-14 from plant viruses (tenuivirus and orthotospovirus), and non-plant viruses (phenuivirus, orthobunyavirus and tenui-like viruses; Fig. 5A–D). Based on the RdRp, three very clear clusters were observed, representing plant virus, non-plant virus and SINV-14 (Fig. 5A). Interestingly, the two tenui-like viruses (WhHV and FCTenv1) were closer to plant tenuiviruses than SINV-14, based on RdRp (Fig. 5A). Moreover, while SINV-14 clearly clustered separately from other groups, based on glycoprotein, nucleocapsid and NS4, this separation was not very clear between plant and non-plant viruses and tenui-like viruses, as observed for RdRp (Fig. 5A–D). We also performed principal component analysis on nucleotide bias obtaining similar results to the codon usage analysis (Fig. 5E–H). Whereas the RdRp cluster was not very clear, based on plant and non-plant viruses, for all other genes SINV-14 clearly separated from any other group (Fig. 5E–H), and again, tenui-like viruses (WhHV and FCTenv1) were closer to other groups than SINV-14.

## Discussion

*S. invicta* is a ground-dwelling ant that feeds on a broad diet that may include plant and insect exudates, prey, and decaying matter. Thus, the opportunities for exchange of virus particles from the environment, whether native or novel, are many. The social nature of ant colonies where exchanges of biological fluids among colony members of all castes and life stages must occur also facilitates distribution of virus within the colony. Distinguishing clearly between different host/microbe relationships and forms of virus transmission (horizontal and vertical) will be challenging with invasive ant systems.

Using a transcriptome approach, we searched for RNA viruses in the SRA data collected from *S. invicta.* Curiously, while a great diversity of positive and negative sense viruses have been reported from ants [9, 10] and other arthropods [2], the *S. invicta* virome has been composed mainly of   ssRNA viruses in the order *Picornavirales*, with no negative sense RNA viruses previously reported. One factor that could be responsible for this bias is the selection of polyadenylated RNA for library preparation in previous studies [11, 30]. The utilization of unbiased library preparation, using ribosomal depletion methods, besides the detection of + ssRNA viruses, has enabled the discovery of many viruses with non-polyadenylated genome, especially within order *Bunyavirales* [2]. The fact of some of the libraries used here were prepared using this approach [36] allowed us to characterize the first bunyavirus reported from ants. In addition, we also reported for the first time another negative sense virus within the order *Mononegavirales* associated with *S. invicta*. Although we did not perform any amplification step of viruses genomes characterized here, the presence of untruncated ORFs carrying intact functional domains (Additional file 3: Table S3), the high abundance of viral reads, and the similar organization and genomes sizes compared to other closely related viruses strongly suggest that we obtained the correct full-length or near full-length genomes sequences (Fig. 1). The only exception is SINaDNV, due to the linear single strand DNA genome, only active transcriptional units were sampled using our transcriptome approach and further investigation is needed to reveal its full-length genome structure.

Our analysis of RNA obtained from geographically and temporally different samples suggests shifts in the virome of native and invasive fire ants [11]. In established exotic populations of the *S. invicta* Yang et al. [21] identified SINV-1 and SINV-2 and hypothesized that while these two viruses may persist, the more virulent virus, SINV-3 arrived with founders but caused high host mortality resulting in individual carriers of the virus being rapidly eliminated. Nine additional viruses were identified in *S. invicta* RNA samples from the native South American range of the species [11]. The viruses described here show distribution and composition that varied according to geographic location and *S. invicta* stage (Tables 1 and 2). To date, four of five viruses previously reported associated with *S. invicta* in introduced areas have also been described to occur in its native origin [11, 21]. Although viral diversity associated with *S. invicta* has been well studied in Argentina, the viruses reported in this study have not been detected there yet [11, 30], suggesting that these viruses may not be present in the sampled area in Argentina, and even that new host-virus associations may have occurred in introduced areas.

While the origins of viruses that replicate in plant and insect vectors remains unknown, such as those within order *Bunyavirales* and *Mononegavirales*, the discovery of possible intermediate forms has been suggested [2, 51]. Whereas tenuiviruses are known to be plant viruses that replicate in the insect vector tenui-like viruses have been reported from non-plant vectors. Li, Shi [2] reported the first tenui-like virus, *Horsefly horwuvirus* (virus WhHV), associated with a pool of horseflies (family *Tabanidae*), and proposed that it may represent a transitional form between plant-infecting virus and arthropod-specific viruses. In addition, a partial genome sequence of another tenui-like, FCTenV1, has been reported associated to *Culex annulirostris* [51]. While these two tenui-like viruses have been reported associated with flies (Order Diptera), we characterized a new tenui-like virus sequence closely related to the FCTenV1, associated with *S. invicta* transcriptome (Order Hymenoptera). Interestingly, in contrast with WhHV, which lacks an ambisense coding strategy, the SINV-14 genome sequence exactly mirrors the genomic structure of typical plant tenuivirus, predicted to encode proteins using ambisense strategy, and also has the conserved sequences at the ends of all four segments identical to those found in tenuivirus genomes (Fig. 1A, B). While these virus sequences may represent different steps of transitional viruses forms between tenui-infecting plant and those insects-specific viruses, the direction of the process, whether they come from plant to insect or otherwise, remains unknown.

Phylogenetic incongruence observed between the nucleocapsid protein compared to the other proteins strongly suggests that SINV-14 may have a recombinant/reassortment origin, where the RNA3 or part of it was acquired from a divergent phenuivirus. While it could be argued that this might be an artefact due to assembling a segmented genome using a transcriptome approach, the fact that we did not find any other contigs related to phenuiviruses, the high read abundance, the constant association between these four segments across different libraries and the presence of the conserved tenuivirus sequence located at the ends of genome segments (Fig. 1B), suggest that they are part of a unique genome, rather than being an artefact. In addition, phylogenetic congruence among different segments indicate that they are in an intimate codivergence process (Additional file 5: Figure S1).

Valles et al. [52] using an expressed sequence tag (EST) library from *S. invicta*, detected a short sequence (approximately 750 nt, GenBank access: EF409991) related to plant tenuiviruses. Further identity analysis showed that sequence is 99.8% identical to SINV14 RNA4. They suggested that the sequence would be likely a contamination due to the ant diet feeding either plant or infected insect. Tenuiviruses are typical plant viruses that replicate in the insect vector [53, 54], and the high abundance of SINV-14 compared to housekeeping genes is strong evidence of active replication in *S. invicta*. Furthermore, the highest virus abundance was found in a sample prepared from a dissected ant brain, which rules out the possibility of contamination due to feeding on plants or association with insect vectors infected with a tenuivirus. The Maize stripe virus (MSpV), a typical plant tenuivirus, was detected in the brain of its vector *Peregrinus maidis*, providing evidence of replication of tenuiviruses in this tissue [55]. Tenuiviruses replicate in diverse tissues of their insect vectors and are transovarially transmitted between generations suggesting that SINV-14, and other tenui-like viruses, could be sustained through vertical transmission in their insect host [54]. Additionally, significant asymmetric abundance sequences of different components of SINV-14 suggest a very specific and active interaction. Asymmetric accumulation in multipartite viruses has been shown and seems to be common trait, shared by RNA and DNA multipartite viruses infecting plants and animals [56, 57]; this has been suggested to be involved in control of gene expression allowing fast virus adaptation [58]. Although this has not yet been shown for any phenuivirus and may be host dependent [56], our results suggest that this may occur, at least for SINV-14, and more experiments will be necessary to confirm.

While SINV-14 is evolutionarily closely related to insect tenui-like viruses and plant tenuivirus, the synonymous codon usage and dinucleotide analyses demonstrate a distinct compositional bias compared to FCTenV1 and WhHV, and all other viruses analyzed here, indicating that the virus may be actively replicating in ants rather than plants and other insects. The active replication may have driven the virus genome to distinct compositional bias at the nucleotide level, while maintaining protein integrity at the amino acid level and close relationship with those of other tenui-like viruses, as observed through phylogenetic analysis. The fact that most sequences examined here are probably from viruses that replicate in plant or vertebrate hosts and also in the insect vector could be the reason driving such difference between SINV-14, most likely associated only with ant, compared to other viruses. Furthermore, phylogenetic congruence across different segments that mirror the genetic structure of invasive *S. invicta*, suggest an intimate and long-term codivergence process between virus-host.

We presented strong evidence that the sequences from SINV-14 may be from a virus that has been associated long-term and may actively replicate in ants. However, the possibility of this virus, and other tenui-like viruses, replicating in plants is unknown. The presence of the protein carrying an NS4 domain, that is related to cell-to-cell and long-distance movement in plants [59] in insect viruses is puzzling. Solenopsis invicta virus 14 NS4 is highly divergent showing 19.85 to 24.4% of identity compared to other tenuivirus (Additional file 6: Figure S2). In addition, mutation in most sites associated with cell-to-cell and long-distance movement (Additional file 6: Figure S2), suggests that this protein might have lost the capacity to move viral genome in plant, whereas its maintenance in insect viruses may be related to another role acquired through functional diversification. The possible function of the plant virus movement protein from insect viruses is of significant interest, and its role in insects and plants remains to be addressed.

Altogether, based on virus abundance compared to housekeeping genes, abundant viruses with viral reads higher than 0.01%, phylogenetic relationship, complete viral coding sequence regions recovered, and compositional bias for SINV-14, our results suggest that four out five viruses reported here, those being SINV-14, SINV-15, SINV-16 and SINV-17 are truly replicating in *S. invicta*. Our results suggest fluid shifts in the virome of this invasive species. Further research describing this virome in native and invasive regions and ecosystems could provide insight on virus evolution and invasion mechanics.

## Conclusions

The present study expands our knowledge about viral diversity associated with *S. invicta* in introduced areas. In addition to revealing new virus-host interactions, contributing to better understanding of viral evolutionary history and emergence, the discovery of new viruses expands the range of agents with potential to be used in biocontrol programs, which will require further biological characterization. By understanding these interactions, we may be better equipped to cope with ongoing global changes and introductions of invasive organisms and their associated viruses.

## Supplementary information

**Additional file 1**. ** Table S1**: Descriptive statistics of fire ant transcriptome datasets analyzed in this study.

**Additional file 2**. ** Table S2**: Open reading frames (ORFs) characterization from putative new viruses based on ORFinder followed by BLASTp analyses, as implemented at National Center for Biotechnology Information Search database (NCBI).

**Additional file 3**. ** Table S3**: Viral conserved domains detected in ORFs from putative new viruses based on Conserved Domains Database (CDD).

**Additional file 4**. ** Table S4**: Data set used in the compositional analyses.

**Additional file 5**. **Figure S1**: Neighbor-Joining phylogenetic tree (1000 bootstrap replications) showing the clustering of the SINV-14 segments. Isolates are highlighted according to sampled place as indicated at the bottom of the figure.

**Additional file 6**. ** Figure S2**: Alignment of non-structural protein 4 (NS4) amino acid sequence of SINV-14 with those from typical plant tenuiviruses. Positions with single conserved amino acid are highlighted in black, groups of similar properties are highlighted in light gray and positions in SINV-14 that shares the same amino acid with at least one plant tenuivirus sequence, are highlighted in dark gray. Sites containing residues essentials for cell-to-cell movement and long-distance movement are indicated by asterisk and filled circle, respectively. These sites were mapped using Maize stripe virus (MSpV) as a model (59). Matrix of pairwise identity of NS4 amino acid sequence are shown at the bottom of the figure. Multiple sequence alignment and pairwise identity were generated in Clustal O v. 1.2.4. European wheat striate mosaic virus (EWSMV), MN044345; Rice hoja blanca virus (RHBV), MG566077; Echinochloa hoja blanca virus (EHBV), L48441; Rice stripe virus (RSV), D10979; Maize stripe virus (MSpV), L13438.

## Data Availability

Consensus virus genome sequences were deposited in GenBank under accession numbers: MT860232 to MT860267.

## Abbreviations

U.S.: United States
+ ssRNA: Positive-sense single-strand RNA
− ssRNA: Negative-sense single-stranded RNA
dsRNA: Double-strand RNA
ssDNA: Single-strand DNA
SINV-1: Solenopsis invicta virus 1
SINV-2: Solenopsis invicta virus 2
SINV-3: Solenopsis invicta virus 3
SINV-6: Solenopsis invicta virus 6
SINV-14: Solenopsis invicta virus 14
SINV-15: Solenopsis invicta virus 15
SINV-16: Solenopsis invicta virus 16
SINV-17: Solenopsis invicta virus 17
SINaDNV: Solenopsis invicta-associated densovirus
NfV-1: Nylanderia fulva virus 1
CDD: Conserved Domain Database
SRA: Sequence Read Archive
cox1: Cytochrome c oxidase subunit I
*rpl18*: Ribosomal protein L18
*eif1-beta*: Translation elongation factor 1
SCU: Synonymous codon usage
PCA: Principal component analysis
RdRp: RNA-dependent RNA polymerase
NS4: Non-structural protein 4 protein
ORF: Open reading frame
FCTenV1: Fitzroy Crossing tenui-like virus 1
EWSMV: European wheat striate mosaic virus
WhHV: Wuhan horsefly virus
EST: Expressed sequence tag
MSpV: Maize stripe virus

## References

[1] Stork NE (2018). How many species of insects and other terrestrial arthropods are there on Earth?. Annu Rev Entomol.

[2] Li CX, Shi M, Tian JH, Lin XD, Kang YJ, Chen LJ (2015). Unprecedented genomic diversity of RNA viruses in arthropods reveals the ancestry of negative-sense RNA viruses. Elife.

[3] Shi M, Lin X-D, Tian J-H, Chen L-J, Chen X, Li C-X (2016). Redefining the invertebrate RNA virosphere. Nature.

[4] Käfer S, Paraskevopoulou S, Zirkel F, Wieseke N, Donath A, Petersen M, et al. Re-assessing the diversity of negative strand RNA viruses in insects. PLoS Pathog. 2019;15(12). 10.1371/journal.ppat.1008224 PMC6932829 31830128

[5] Shi M, Neville P, Nicholson J, Eden JS, Imrie A, Holmes EC (2017). High-resolution metatranscriptomics reveals the ecological dynamics of mosquito-associated RNA viruses in western Australia. J Virol.

[6] Atoni E, Zhao L, Karungu S, Obanda V, Agwanda B, Xia H (2019). The discovery and global distribution of novel mosquito-associated viruses in the last decade (2007–2017). Rev Med Virol.

[7] Faizah AN, Kobayashi D, Isawa H, Amoa-Bosompem M, Murota K, Higa Y, et al. Deciphering the virome of *Culex vishnui* subgroup mosquitoes, the major vectors of japanese encephalitis, in Japan. Viruses. 2020;12(3). 10.3390/v12030264 PMC7150981 32121094

[8] Pettersson JH, Shi M, Eden JS, Holmes EC, Hesson JC. Meta-transcriptomic comparison of the RNA viromes of the mosquito vectors *Culex pipiens* and *Culex torrentium* in Northern Europe. Viruses. 2019;11(11). 10.3390/v11111033 PMC6893722 31698792

[9] Kleanthous E, Olendraite I, Lukhovitskaya NI, Firth AE (2019). Discovery of three RNA viruses using ant transcriptomic datasets. Adv Virol.

[10] Dhaygude K, Johansson H, Kulmuni J, Sundström L. Genome organization and molecular characterization of the three Formica exsecta viruses-FeV1, FeV2 and FeV4. PeerJ. 2019;20(6). 10.7717/peerj.6216 PMC6387575 30809424

[11] Valles SM, Rivers AR (2019). Nine new RNA viruses associated with the fire ant *Solenopsis invicta* from its native range. Virus Genes.

[12] Lacey LA, Grzywacz D, Shapiro-Ilan DI, Frutos R, Brownbridge M, Goettel MS (2015). Insect pathogens as biological control agents: back to the future. J Invertebr Pathol.

[13] Chen X, Gonçalves MA (2016). Engineered viruses as genome editing devices. Mol Ther.

[14] Rode NO, Estoup A, Bourguet D, Courtier-Orgogozo V, Débarre F (2019). Population management using gene drive: molecular design, models of spread dynamics and assessment of ecological risks. Conserv Genet.

[15] Porter SD, Savignano DA (1990). Invasion of polygyne fire ants decimates native ants and disrupts arthropod community. Ecology.

[16] Pimentel D, Zuniga R, Morrison D (2005). Update on the environmental and economic costs associated with alien-invasive species in the United States. Ecol Econ.

[17] Callcott A-MA, Collins HL. Invasion and range expansion of imported fire ants (Hymenoptera: Formicidae) in North America from 1918–1995. The Florida Entomologist. 1996;79(2):240–51.

[18] Ascunce MS, Yang C-C, Oakey J, Calcaterra L, Wu W-J, Shih C-J (2011). Global invasion history of the fire ant *Solenopsis invicta*. Science.

[19] Shoemaker DD, Ahrens M, Sheill L, Mescher M, Keller L, Ross KG (2003). Distribution and prevalence of *Wolbachia* Infections in native populations of the fire ant *Solenopsis invicta* (Hymenoptera: Formicidae). Environ Entomol.

[20] Bouwma AM, Ahrens ME, DeHeer CJ, DeWayne SD (2006). Distribution and prevalence of *Wolbachia* in introduced populations of the fire ant *Solenopsis invicta*. Insect Mol Biol.

[21] Yang C-C, Yu Y-C, Valles SM, Oi DH, Chen Y-C, Shoemaker D (2010). Loss of microbial (pathogen) infections associated with recent invasions of the red imported fire ant *Solenopsis invicta*. Biol Invasions.

[22] Porter SD, Fowler HG, Mackay WP (1992). Fire ant mound densities in the United States and Brazil (Hymenoptera: Formicidae). J Econ Entomol.

[23] Morrison LW, Porter SD, Daniels E, Korzukhin MD (2004). Potential global range expansion of the invasive fire ant, *Solenopsis invicta*. Biol Invasions.

[24] Drees BM, Calixto AA, Nester PR (2013). Integrated pest management concepts for red imported fire ants *Solenopsis invicta* (Hymenoptera: Formicidae). Insect Science.

[25] Valles SM, Porter SD, Choi M-Y, Oi DH (2013). Successful transmission of Solenopsis invicta virus 3 to *Solenopsis invicta* fire ant colonies in oil, sugar, and cricket bait formulations. J Invertebr Pathol.

[26] Oi D, Valles S, Porter S, Cavanaugh C, White G, Henke J. Introduction of fire ant biological control agents into the Coachella Valley of California. Florida Entomologist. 2019;102(1):284–6, 3.

[27] Valles SM, Strong CA, Dang PM, Hunter WB, Pereira RM, Oi DH (2004). A picorna-like virus from the red imported fire ant, *Solenopsis invicta*: initial discovery, genome sequence, and characterization. Virology.

[28] Valles SM, Hashimoto Y (2009). Isolation and characterization of Solenopsis invicta virus 3, a new positive-strand RNA virus infecting the red imported fire ant *Solenopsis invicta*. Virology.

[29] Valles SM, Strong CA, Hashimoto Y (2007). A new positive-strand RNA virus with unique genome characteristics from the red imported fire ant *Solenopsis invicta*. Virology.

[30] Valles SM, Porter SD, Calcaterra LA. Prospecting for viral natural enemies of the fire ant *Solenopsis invicta* in Argentina. PLoS One. 2018;13(2). 10.1371/journal.pone.0192377 PMC5821328 29466388

[31] Allen ML (2020). Near-complete genome sequences of new strain of *Nylanderia Fulva Virus 1* from *Solenopsis invicta*. Microbiol Resour Announc.

[32] Valles SM, Shoemaker D, Wurm Y, Strong CA, Varone L, Becnel JJ (2013). Discovery and molecular characterization of an ambisense densovirus from South American populations of *Solenopsis invicta*. Biol Control.

[33] Manfredini F, Shoemaker D, Grozinger CM (2015). Dynamic changes in host-virus interactions associated with colony founding and social environment in fire ant queens (*Solenopsis invicta*). Ecol Evol.

[34] Hsu H-W, Chiu M-C, Shoemaker D, Yang C-CS. Viral infections in fire ants lead to reduced foraging activity and dietary changes. Sci. Rep. 2018;8(1):13498. 10.1038/s41598-018-31969-3 PMC6131164 30202033

[35] Valles SM (2012). Positive-strand RNA viruses infecting the red imported fire ant, *Solenopsis invicta*. Psyche.

[36] Allen ML, Rhoades JH, Sparks ME, Grodowitz MJ. Differential gene expression in red imported fire ant (*Solenopsis invicta*) (Hymenoptera: Formicidae) larval and pupal stages. Insects. 2018;9(4). 10.3390/insects9040185 PMC6315859 30563147

[37] Morandin C, Tin MMY, Abril S, Gómez C, Pontieri L, Schiøtt M (2016). Comparative transcriptomics reveals the conserved building blocks involved in parallel evolution of diverse phenotypic traits in ants. Genome Biol.

[38] Fontana S, Chang N-C, Chang T, Lee C-C, Dang V-D, Wang J (2020). The fire ant social supergene is characterized by extensive gene and transposable element copy number variation. Mol Ecol.

[39] Kumar S, Stecher G, Tamura K. MEGA7: molecular evolutionary genetics analysis version 7.0 for bigger datasets. Mol Biol Evol. 2016;33(7):1870–4. 10.1093/molbev/msw054 PMC8210823 27004904

[40] Ronquist F, Teslenko M, van der Mark P, Ayres DL, Darling A, Höhna S, et al. MrBayes 3.2: efficient Bayesian phylogenetic inference and model choice across a large model space. Syst Biol. 2012;61(3):539–42. Epub 2012/02/22. 10.1093/sysbio/sys029 PMC3329765 22357727

[41] Bewick V, Cheek L, Ball J (2004). Statistics review 10: further nonparametric methods. Critical Care (London, England).

[42] De Mendiburu F, Yaseen M. Statistical procedures for agricultural research. R package version 140. https://myaseen208.github.io/agricolae/2020.

[43] Di Giallonardo F, Schlub TE, Shi M, Holmes EC (2017). Dinucleotide composition in animal RNA viruses is shaped more by virus family than by host species. J Virol.

[44] Kapoor A, Simmonds P, Lipkin WI, Zaidi S, Delwart E (2010). Use of nucleotide composition analysis to infer hosts for three novel picorna-like viruses. J Virol.

[45] Elek A, Kuzman M, Vlahovicek K. coRdon: codon usage analysis and prediction of gene expressivity. R package version 170. https://github.com/BioinfoHR/coRdon2020.

[46] Charif D, Lobry JR. SeqinR 1.0-2: a contributed package to the R project for statistical computing devoted to biological sequences retrieval and analysis. In: Bastolla U, Porto M, Roman HE, Vendruscolo M, editors. Structural Approaches to Sequence Evolution: Molecules, Networks, Populations. Berlin, Heidelberg: Springer Berlin Heidelberg; 2007. p. 207–32.

[47] Kassambara A, Mundt F. factoextra: extract and visualize the results of multivariate data analyses. R package version 107. http://www.sthda.com/english/rpkgs/factoextra2020.

[48] Vasilakis N, Forrester NL, Palacios G, Nasar F, Savji N, Rossi SL (2013). Negevirus: a proposed new taxon of insect-specific viruses with wide geographic distribution. J Virol.

[49] Toriyama S, Kimishima T, Takahashi M, Shimizu T, Minaka N, Akutsu K (1998). The complete nucleotide sequence of the rice grassy stunt virus genome and genomic comparisons with viruses of the genus *Tenuivirus*. J Gen Virol.

[50] Valles SM, Oi DH, Becnel JJ, Wetterer JK, LaPolla JS, Firth AE (2016). Isolation and characterization of Nylanderia fulva virus 1, a positive-sense, single-stranded RNA virus infecting the tawny crazy ant, *Nylanderia fulva*. Virology.

[51] Williams SH, Levy A, Yates RA, Somaweera N, Neville PJ, Nicholson J (2020). The diversity and distribution of viruses associated with *Culex annulirostris* mosquitoes from the Kimberley region of western Australia. Viruses.

[52] Valles SM, Strong CA, Hunter WB, Dang PM, Pereira RM, Oi DH (2008). Expressed sequence tags from the red imported fire ant, *Solenopsis invicta*: annotation and utilization for discovery of viruses. J Invertebr Pathol.

[53] Falk BW, Tsai JH (1998). Biology and molecular biology of viruses in the genus *Tenuivirus*. Annu Rev Phytopathol.

[54] Liu W, Hajano JU, Wang X (2018). New insights on the transmission mechanism of tenuiviruses by their vector insects. Curr Opin Virol.

[55] Nault LR, Gordon DT (1988). Multiplication of Maize Stripe Virus in Peregrinus maidis. Phytopathology.

[56] Sicard A, Yvon M, Timchenko T, Gronenborn B, Michalakis Y, Gutierrez S, et al. Gene copy number is differentially regulated in a multipartite virus. Nat Commun. 2013;4(2248). 10.1038/ncomms3248 23912259

[57] Wu B, Zwart MP, Sánchez-Navarro JA, Elena SF (2017). Within-host evolution of segments ratio for the tripartite genome of Alfalfa mosaic virus. Sci. Rep..

[58] Zwart MP, Elena SF. Modeling multipartite virus evolution: the genome formula facilitates rapid adaptation to heterogeneous environments. Virus Evol. 2020;6(1). 10.1093/ve/veaa022 PMC7206449 32405432

[59] Zhang C, Pei X, Wang Z, Jia S, Guo S, Zhang Y (2012). The *Rice stripe virus* pc4 functions in movement and foliar necrosis expression in *Nicotiana benthamiana*. Virology.

