# Supplementary figures and images for: Ever-increasing viral diversity associated with the red imported fire ant Solenopsis invicta (Formicidae: Hymenoptera)
Source: Virol J. 2021 Jan 6;18:5. doi: 10.1186/s12985-020-01469-w (PMC7788728; doi:10.1186/s12985-020-01469-w)

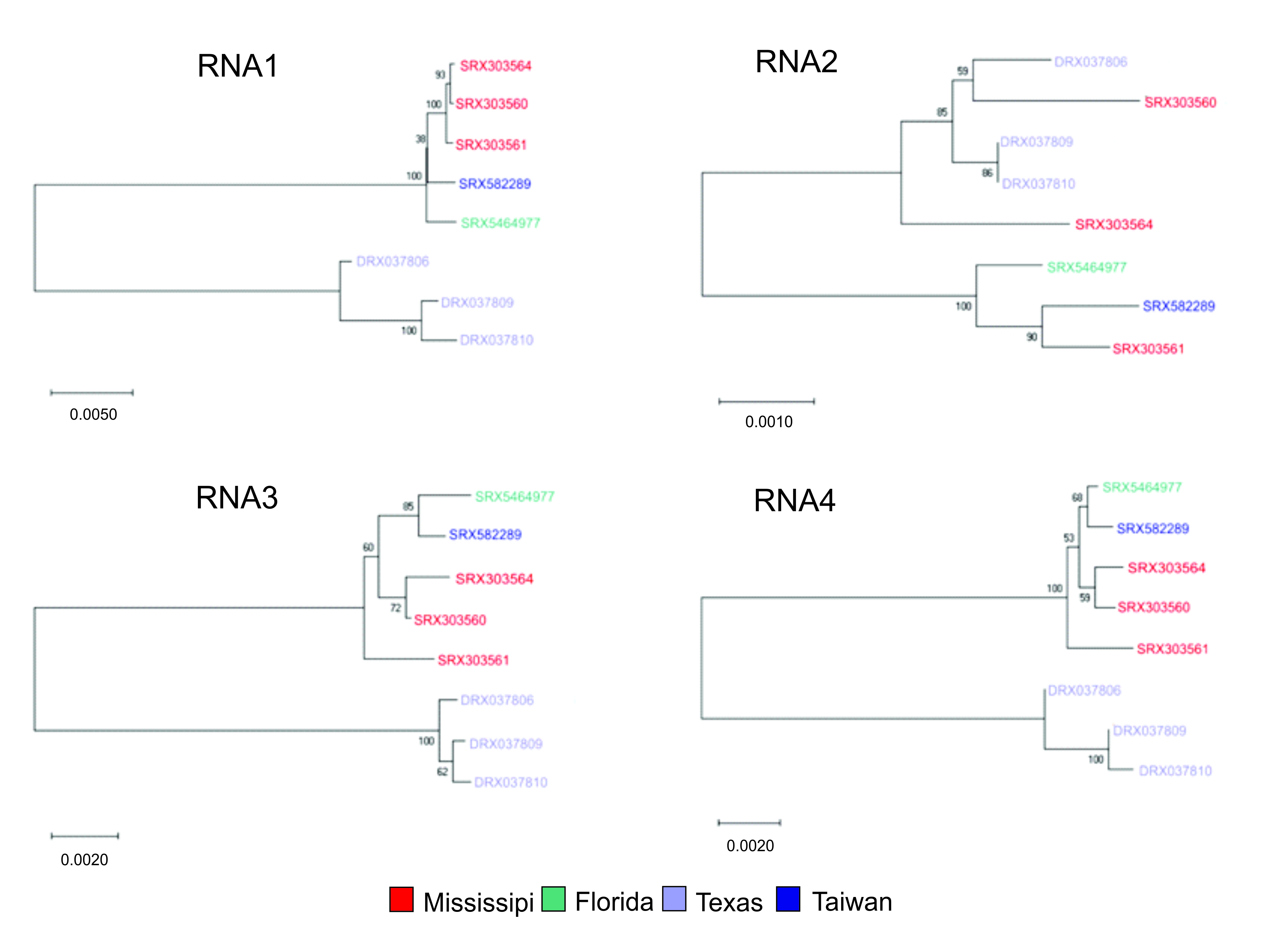

Supplement: Supplementary file 5 — Additional file 5. Figure S1: Neighbor-Joining phylogenetic tree (1000 bootstrap replications) showing the clustering of the SINV-14 segments. Isolates are highlighted according to sampled place as indicated at the bottom of the figure. [file 12985_2020_1469_MOESM5_ESM.tif]

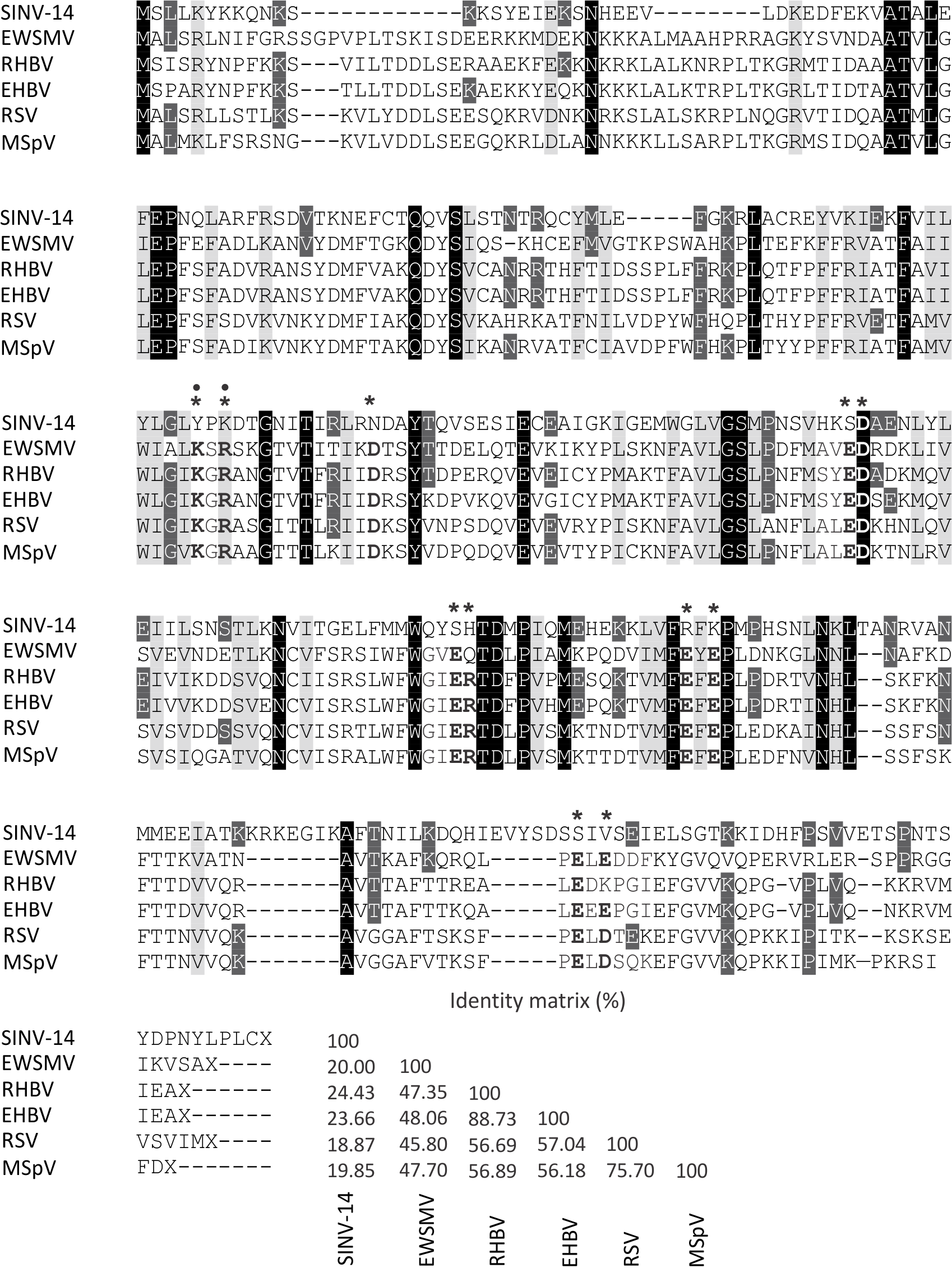

Supplement: Supplementary file 6 — Additional file 6. Figure S2: Alignment of non-structural protein 4 (NS4) amino acid sequence of SINV-14 with those from typical plant tenuiviruses. Positions with single conserved amino acid are highlighted in black, groups of similar properties are highlighted in light gray and positions in SINV-14 that shares the same amino acid with at least one plant tenuivirus sequence, are highlighted in dark gray. Sites containing residues essentials for cell-to-cell movement and long-distance movement are indicated by asterisk and filled circle, respectively. These sites were mapped using Maize stripe virus (MSpV) as a model (59). Matrix of pairwise identity of NS4 amino acid sequence are shown at the bottom of the figure. Multiple sequence alignment and pairwise identity were generated in Clustal O v. 1.2.4. European wheat striate mosaic virus (EWSMV), MN044345; Rice hoja blanca virus (RHBV), MG566077; Echinochloa hoja blanca virus (EHBV), L48441; Rice stripe virus (RSV), D10979; Maize stripe virus (MSpV), L13438. [file 12985_2020_1469_MOESM6_ESM.tif]
